# Comparative virulotyping and phylogenomics of *Escherichia coli *isolates from urine samples of men and women suffering urinary tract infections

**DOI:** 10.22038/ijbms.2018.28360.6880

**Published:** 2019-02

**Authors:** Hamid Staji, Maryam Rassouli, Samaneh Jourablou

**Affiliations:** 1 Department of Pathobiology, Faculty of Veterinary Medicine, Semnan University, Semnan, Iran; 2 Graduated student (DVM), Faculty of Veterinary Medicine, Semnan University, Semnan, Iran

**Keywords:** Female, Male, Phylogeny, Urinary tract infections, Uropathogenic Escherichia coli, Virulence

## Abstract

**Objective(s):**

*Escherichia coli* strains are common pathogens that can cause urinary tract infections (UTI). This study aimed to assess *E. coli* phylogroups and virulence types in male and female UTI patients.

**Materials and Methods:**

In the present study, 160 uropathogenic* E. coli* (UPEC) isolates (from both sexes) were assigned to phylogroups/types and some extraintestinal virulence factors were detected within them by multiplex-PCR.

**Results:**

The isolates from women and men were predominantly distributed within phylogroup B_2_ and D, respectively. The presence of D_2_ phylotype was higher in men isolates than women, significantly (*P*=0.045). In male isolates *papEF* and *sfa/focDE* are more prevalent in B_2_ group than D, significantly (*P*=0.048; *P*=0.035). The prevalence of *hly* in B_2_ group is significantly higher than D (*P*=0.034) in female isolates.

**Conclusion:**

This study highlighted different features of *E. coli* genotypes from phylogenetic and virulence point of view implicated in UTI’s in both human genders.

## Introduction

*Escherichia coli* (*E. coli*) strains belonging to *enterobacteriaceae* family are normal habitant of gastro-intestinal tract in the wide range of warm-blooded hosts. Many *E. coli* strains are harmless while some are pathogenic, meaning that they can cause illness, either diarrhea or infections in extraintestinal sites such as urinary tract infections (UTI) in human (1, 2). After respiratory infections, the UTI’s are the most common infectious problem in human resulting in serious sequelae if remain untreated and Uropathogenic *E*. *coli* (UPEC) is responsible for more than %80 of UTI’s (3, 4). *E. coli* strains based on some genetic markers (*chuA*, *yjaA*, and *tspE4.C2*) are divided into four main phylogenetic groups A, B_1_, B_2_ and D consisting of seven phylotypes: phylotypes A_0_ and A_1_ (group A); B_1_ (group B_1_); B_22_ and B_23_ (group B_2_); D_1_ and D_2_ (group D) (5). It is believed that groups B_2_ and D include the majority of virulent extraintestinal *E. coli*, whilst groups A and B_1_ primarily represent commensal characteristics (6, 7). On the other hand, UPEC strains have multiple virulence factors (VFs) that confer the potential for pathogenicity, invasion, and colonization of urinary tract sites such as P and S fimbriae, afimbrial adhesion molecules and hemolysin through pathogenicity islands (8). There are some evidences demonstrating that sex and gender can imply an important role in the formation of microbiota of human flora sites. Pathogenic species and phenotypic characteristics causing UTI’s are affected by such factors like gender (9-12). Accordingly, in the present study, relationships between the presence of selected virulence genes and the phylogroup of *E. coli* strains implicated in men and women UTI cases were evaluated to assess if there are genotypic differences between *E. coli* isolates in these two hosts.

## Materials and Methods

***Isolation of E. coli strains from patients with UTI***

One hundred and sixty *E. coli* strains were isolated from patients (25 - 45 years old/both sexes) suspected to suffering UTI based on urologist’s diagnosis referring to diagnostic laboratories according to the protocol described by Bonadio *et al* (13). Then stock cultures were prepared from the *E. coli* isolates and stored in Luria-Bertani broth (Sigma-Aldrich) with 15% (v/v) glycerol at -20 ^°^C until genotyping. 

***DNA extraction, phylogenetic and virulence genotyping***

Genomic DNA was extracted from *E. coli* strains with the rapid one-step extraction (ROSE) method (14) based on alkaline lysis of bacteria and phylogenetic group and phylotype of isolates were identified using a modified Triplex PCR-based assay optimized for detection of *chuA*, *yjaA*, and *tspE4.C2* gene markers as previously described by Staji (15). Finally, the detection of four extraintestinal putative virulence genes (*hly*, *iucD*, *sfa/focDE *and *pap*) was carried out using a modified Tetraplex-PCR program as described by Staji *et al* previously (8). The primers sequences used in this study are present in Table 1. 

***Statistical analysis***

Differences in frequencies of phylogenetic groups and virulence markers among men and women isolates was evaluated using chi-squared tests (x^2^) on contingency tables and Fisher’s exact test with a significance level of *P*=0.05, using SPSS (version 21) software.

## Results

A total of 160 *E. coli* isolates, 100 from females and 60 from male patients, were obtained from 240 urine samples (66.6%) of human cases suffering UTI, confirming that *E. coli* is dominant agent in development of urinary tract infections. All phylogenetic groups and phylotypes were found among isolates from both sex individuals in the general population, except for A_0_ phylotype which was absent in *E. coli* isolates from females (Table 2). Distribution of the phylogenetic group’s diﬀered depending on the host sexuality. In females (n=100 strains), 46% of isolates belonged to group B_2_, 33% to group D, 13% to group A, and 8% to group B_1_. In male patients (n=60 strains), phylogroup B_2_ was predominant (48.3%) followed by phylogroups D (25%), A (20%) and group B_1_ (6.7%). Distribution of *E. coli *phylotypes are presented in Table 2 in detailed. Statistical analyses showed that there is no significant differences between distributions of phylogenetic groups in male and female isolates. Comparison of prevalence of phylotypes between both sexes shows that D_2_ phylotype is more prevalent in male isolates than females (*P*=0.045), significantly. 

The detection of virulence factors (*hly*; *iucD*; *pap*; *sfa/focDE*) using Multiplex-PCR revealed that 158 (98.75%) of all strains (n=160) were positive for at least 1 of the virulence genes tested; of these strains, 100 (100%) were female and 58 (96.6%) were male isolates. In the strains isolated from female patients, *iucD* and *sfa/focDE* were the most common virulence genes identified (47% and 43%, respectively), followed by *hly* (15%) and *pap* (13%). The only significant difference in the prevalence of virulence genes among female phylogroups showed that *hly* was significantly more prevalent in B_2_ compared to D phylogroup (*P*=0.034).

In the male strains, *iucD*, *pap* and *sfa/focDE* were the most common virulence genes detected, present in 36%, 16% and 13% of isolates, respectively, and followed by *hly* (4%). In the *E. coli* isolates related to male patients *sfa/focDE* and *pap* (*P*=0.035 and *P*=0.048, respectively) were significantly more prevalent in B_2_ phylogroup compared to D. The prevalence of virulence genes in *E. coli* phylogroups and phylotypes of human UTI’s are present in Table 2. 

Statistical analyses on the prevalence of virulence factors between *E. coli* isolates, stratifying by host gender, showed that *pap* is more prevalent in male isolates (*P*=0.035) whereas *sfa/focDE* is more prevalent in female strains (*P*=0.006), significantly.

## Discussion

*E. coli* is the predominant etiology of UTI and in this study a total of 160 *E. coli* isolates collected from the urine samples of female (62.5%) and male (37.5%) patients suffering from UTI caused by *E. coli* were examined to determine the phylogenetic group and phylotypes of each strains and the presence of the four extraintestinal pathogenic *E. coli* (ExPEC) related virulence genes. Finally, the distribution of phylogenetic characteristics and mentioned virulence genes were compared in *E. coli* strains of both sexes to investigate the diﬀerences in genotypes between the two sources. 

**Table 1:** Polymerase chain reaction primers used to detect phylogenetic groups and extraintestinal virulence factors genes of E. coli in samples collected from male and female patients suffering UTI

Gene Name	Primer sequence (5’ to 3’)	Product size (bp)	Reference (5, 23)
***chuA***	GACGAACCAACGGTCAGGAT	279	Carlos et al., (2010)
	TGCCGCCAGTACCAAAGACA		
***yjaA***	TGCCGCCAGTACCAAAGACA	211	,,
	ATGGAGAATGCGTTCCTCAAC		
***tspE4.C2***	GAGTAAGTTCGGGGCATTCA	152	,,
	CGCGCCAACAAAGTATTACG		
***hly***	AACAAGGATAAGCACTGTTCTGGCT	1177	Adib et al., (2014)
	ACCATATAAGCGGTCATTCCCGTCA		
***iucD***	TACCGGATTGTCATATGCAGACCGT	602	,,
	AATATCTTCCTCCAGTCCGGAGAAG		
***Pap***	GCAACAGCAACGCTGGTTGCATCA	336	,,
	AGAGAGAGCCACTCTTATACGGACA		
***Sfa/focDE***	CTCCGCAGAACTGGGTGCATCTTA	410	,,
	CGCAGGAGTAATTACAAACCTGGCA		

**Table 2:** Frequency of *E. coli* phylogenetic Types (PT) and virulence genes (%) in relation to PT among urinary *E. coli* isolates from human female and male UTI cases

Source	PT	No. Strains (%)	Virulence Genes (%)			
			*hly*	*iucD*	*Sfa/focDE*	*pap*
Female UTI	A_0_	0	-	-	-	-
	A_1_	13 (13)	0	6 (12.75)	1 (2.3)	1
	B_1_	8 (8)	1 (6.7)	2 (4.2)	5 (11.6)	1
	B_22_	5 (5)	0	3 (6.4)	1 (2.3)	1
	B_23_	41 (41)	12 (80)	19 (40.5)	23 (53.5)	8
	D_1_	12 (12)	1 (6.7)	5 (10.6)	5 (11.6)	1
	D_2_	21 (21)	1 (6.7)	12 (25.5)	8 (18.6)	1
	Total	100 (100)	15 (100)	47 (100)	43 (100)	13 (100)
Male UTI	A_0_	2 (3.3)	0	0	0	0
	A_1_	10 (16.7)	0	5 (14)	0	0
	B_1_	4 (6.7)	0	2 (5.5)	1 (7.7)	1 (6.2)
	B_22_	3 (5)	0	2 (5.5)	0	1 (6.2)
	B_23_	26 (43.3)	4 (100)	20 (55.5)	11 (84.6)	12 (75)
	D_1_	10 (16.7)	0	5 (14)	0	1 (6.2)
	D_2_	5 (8.3)	0	2 (5.5)	1 (7.7)	1 (6.2)
	Total	60 (100)	4 (100)	36 (100)	13 (100)	16 (100)

All phylogenetic groups and subgroups were found among isolates from UTI’s and our results show that groups B_2_ (46.8%) and D (30%) are the most prevalent phylogenetic groups in *E. coli* strains implicated in human urinary tract infections (Table 2). These results are in parallel with some phylogenetic epidemiological studies about UPEC, as reported by Staji *et al* (8). It has been demonstrated that ExPEC strains usually belong to groups B_2_ and D. In our study about 23% of isolates belonged to groups A and B_1_. These findings are in accordance with the results of Bingen *et al.* and Pupo *et al.* demonstrating that the route of infection can be intestinal pathogenic *E. coli* and commensal strains (17, 18). Although there are some evidences about the impact of host gender on the pathogenic aspects of *E. coli* in UTIs (12) but there is no data about comparative phylogenomics of this pathogenic agent between male and female patients. The comparison of phylogenetic groups and phylotypes distribution between isolates from male and female cases demonstrated that the only significant difference is in D_2_ phylotype while it was more prevalent in male *E. coli* isolates from male UTI cases. The difference between D_1_ and D_2_ phylotypes is harboring *tspE4.C2* genetic marker by D_2_ which its role in the pathogenesis is unclear (19). Any way considering *tspE4.C2* as a virulence factor and higher distribution of this element in male UPEC, it can be concluded that UPEC strains in male patients are more virulent than female ones. 

Detection of virulence genes in the strains from UTI’s shows the presence of isolates harboring a combination of *hly*, *iucD*, *pap* and *sfa/focDE *genes, which could implicate the presence of Pathogenicity Islands (PAI’s) – typical chromosomal traits of the UPEC strains (8, 20). As expected, the majority of urinary strains (98.7%) harbored extraintestinal virulence factor-encoding genes and only 2 isolates (1.2%) in our study were negative for the mentioned virulence genes. Previous studies have assessed the prevalence of *E. coli* strains showing multiple virulence factors patterns implicated in extra-intestinal infections and they have concluded that this prevalence is strongly associated with strains belonging to phylogroup B_2_ (21, 22). Comparison of the virulence factors distribution in *E. coli* phylogroups from male and female’s demonstrated that in women isolates *hly* is significantly more prevalent in B_2_ group versus D, while in men isolates *sfa/focDE *and *pap* genes are more prevalent in B_2_ versus D, significantly. UPEC possess a diverse array of adherence factors and fimbriae such as type 1, P, F1C/S and AFA fimbriae represent the primary mediators for colonization of the urinary tract. An important step in the onset and expansion of UTI is adhesion of *E. coli* to uroepithelial cells by P fimbriae. S fimbriae has also been shown to attach efficiently to the epithelial and endothelial cells of the lower human urinary tract. Studies have shown that *papEF* and *sfa/focDE* are essential for cystitis and/or pyelonephritis (16, 23, 24). In the present study it is observed that *papEF* is more prevalent in male isolates, while in female isolates *sfa/focDE* is dominant, significantly. These results show that in men and women UTI caused by *E. coli*, the type of adhesion and fimbriae may quite be different and this can be because of anatomic differences and fimberial receptors on the epithelial cells of urinary tract. Also the comparison of distribution of these two adhesins in B_2_ and D phylogroups of this both genders shows that there is no difference in the type of adhesins in female isolates, but in male isolates presence of *papEF* and *sfa/focDE* is dominant in B_2_ phylogroup versus D, significantly. These findings confer that UPEC having any type of related adhesins belonging to B_2_ and D groups may be able to onset UTI in women but *E. coli* strains with *pap* and *sfa/focDE *belonging to B_2_ are more prone to induce UTI in men. 

Detection of *iucD* in *E. coli* isolates showed that 52% of tested strains are positive for this virulence factor, and assessment of this VF in different phylogroups showed that 82% of *iucD* are present in B_2_ and D groups. There was not any sex depended differences between distribution of *iucD* prevalence in *E. coli* isolates. About 48% of our isolates are lacking *iucD*, considering that ExPEC strains have evolved multiple strategies for sequestering iron from the host, probably they have other strategies for iron uptake from the host organs (25).

## Conclusion

The results show that phylogroups B_2_ and D, are the major *E. coli* strains causing human urinary tract infections and strains causing such infections in different genders can vary in virulence genotypes, especially in type of adhesins for the colonization and onset of the infection. More work seems to be necessary for full genotyping of *E. coli* strains isolated from both sexes and assessment of antimicrobial resistance profile of such isolates from these two sources. 
